# RETRACTION: All‐Trans Retinoic Acid Enhances Chemosensitivity to 5‐FU by Targeting miR‐378c/E2F7 Axis in Colorectal Cancer

**DOI:** 10.1155/jo/9824048

**Published:** 2026-02-12

**Authors:** Journal of Oncology

RETRACTION: J. Li, Q. Xiang, M. Wang, H. Zhang, and R. Liang, “All‐Trans Retinoic Acid Enhances Chemosensitivity to 5‐FU by Targeting miR‐378c/E2F7 Axis in Colorectal Cancer,” *Journal of Oncology* 2021, no. 1 (2021): 1–10, https://doi.org/10.1155/2021/5338934.

The above article, published online on 21 July 2021 in Wiley Online Library (https://wileyonlinelibrary.com), has been retracted by agreement between the authors and John Wiley & Sons Ltd.

The retraction has been agreed following an investigation into concerns initially raised on PubPeer [1]. The following areas of figure overlap have been identified:

• The first panel in Figure 1D shows substantial overlap with the first panel in Figure 1E.
• The bottom portion of the second panel in Figure 1D appears to overlap with the top portion of the third panel in Figure 1D.
• The second panel in Figure 1D shows substantial similarities with the third panel in the bottom row of Figure 3B in a published article by a different author group involving different experimental targets and cell lines [2].
• The top portion of the first panel in Figure 1E overlaps with the bottom portion of the first panel in the top row of Figure 2D in another published article by a different author group involving different experimental targets and cell lines [3].
• The bottom right portion of the second panel in Figure 1E appears to overlap with the top left portion of the third panel in Figure 1E.
• The third panel in Figure 1E shows substantial similarities with the fourth panel in the bottom row of Figure 3B in [2].
• Numerous portions of the images in Figures 1B, 3D, and 6D also were identified by the publisher as showing significant overlap.

The authors of this article were unable to provide their original raw data and images when asked to address these concerns, and therefore they requested that the article be retracted. Because of the significant duplications identified, the data and conclusions of this article are considered unreliable. Therefore, the article must be retracted.
